# Modified enzyme multiplied immunoassay technique of methotrexate assay to improve sensitivity and reduce cost

**DOI:** 10.1186/s40360-018-0283-5

**Published:** 2019-01-09

**Authors:** Xiaoping Shi, Hui Gao, Zhong Li, Jinghua Li, Yang Liu, Lujuan Li, Qi Zhang

**Affiliations:** 1 Pharmacy Department of Dalian Children’s Hospital, No.154, Zhongshan Road, Dalian City, 116012 Xigang District China; 2 Hematology Ward of Dalian Children’s Hospital, No.154, Zhongshan Road, Dalian City, 116012 Xigang District China

**Keywords:** Methotrexate, Enzyme multiplied immunoassay technique, Sensitivity, Cost reduction

## Abstract

**Background:**

Methotrexate is an important component in many chemotherapy protocols. The blood concentration of Methotrexate is used to determine the regimen of folinic acid. However, the lower limit of Siemens assay kit is 0.30 μmol/L in China. This study extended the limit from 0.3 to 0.05 μmol/L and reduced the test cost by optimizing the parameters of Enzyme Multiplied Immunoassay Technique assay.

**Methods:**

Parameters of Enzyme Multiplied Immunoassay Technique assay were modified to decrease the volume of reagents A and B. Then a standard curve with a new custom set of calibrators was prepared to detect low concentration. Intra-day and inter-day imprecision were assessed by control material and samples. The linearity of the modified assay was verified by analyzing a range of quality controls with known concentration from 0.05 to 1.00 μmol/L. At last, the same samples were tested by modified Enzyme Multiplied Immunoassay Technique assay and Liquid Chromatography-tandem Mass Spectrometry assay respectively. A simple linear regression was performed to verify the validity of the modified Enzyme Multiplied Immunoassay Technique assay.

**Results:**

Intra-day and inter-day imprecision show good reproducibility at all levels (0.05, 0.12, 0.43, 0.82 μmol/L). The linearity equation of modified assay was y = 0.9913x + 0.0046, in which y was the mean of measured concentration and x was the target concentration (R^2^ = 0.9994). In the range of 0.05–10.00 μmol/L, correlation between the Modified assay and Liquid Chromatography-tandem Mass Spectrometry assay was significant (*r* = 0.9968). In the range of 0.30–10.00 μmol/L, the correlation between modified and commercial assays was significant (*r* = 0.9987) as well.

**Conclusions:**

The modified assay enhanced the sensitivity of Siemens VIVA-E to 0.05 μmol/L. In addition, the test number of a reagent Kit increased from 140 to 210. This means the cost of detection was reduced about 30%.

## Background

Methotrexate (MTX) has been used worldwide to treat a broad spectrum of diseases, such as leukemia, non-Hodgkin’s lymphoma, osteosarcoma and other malignancies. When high dose of MTX (HDMTX) is administered intravenously and followed by rescue with folinic acid, it is crucial to monitor the blood concentration of MTX at the fixed time points to determine the optimal dose and regimen of folinic acid. In general, folinic acid rescue is continued until MTX concentration fall below 0.10 μmol/L at 72 h or below 0.05 μmol/L at 96 h after MTX administration [1]. Siemens VIVA-E instrument provides an effective assay for monitoring MTX. However, it has two deficiencies. Firstly, the lower limit of this assay is just 0.30 μmol/L, which is insufficient to meet the diagnostic criterion to terminate folinic acid rescue. Secondly, the cost of MTX assay on Viva-E is so high that hospital’s laboratory could not afford routine test. There were many articles about how to enhance the sensitivity of Enzyme Multiplied Immunoassay Technique (EMIT) assay, but the cost of MTX detection has been ignored [2, 3]. In this study, not only the lower limit of quantitation decreased from 0.30 μmol/L to 0.05 μmol/L, but also the cost of detection was reduced about 30%.

## Methods

### Instrument and reagents

The instrument for MTX detection was Siemens VIVA-E which allows for parameter customization through the availability of open channel. MTX reagents and calibrators were purchased from Siemens (6L119UL).

Three levels of Quality control (QC) material were purchased from Bio-Rad (Irvine, CA), an additional custom QC material at 0.05 μmol/L from Aladdin (Shanghai, China).

MTX method comparison was performed by analyzing the same samples using Liquid Chromatography-tandem Mass Spectrometry (LC-MS/MS) assay (LC-20 AD Liquid Chromatography, and AB SCIEX QTRAP®4500 Mass Spectrometers).

### Specimens

The test samples were obtained from the hematological ward of Dalian children’s hospital. Some tested samples and MTX-free icteric plasma samples were collected for studying on triglyceride and bilirubin interference. There was no intervention in the treatment of patients, and none of patient’s individual information was mentioned.

### Reagent preparation

The Siemens Reagent Kit contains Antibody/Substrate Reagent A, Enzyme Reagent B, Emit Drug Assay Buffer Concentrate, and Emit Methotrexate Calibrators. Reagent A and B were reconstituted with 3.0 mL deionized water, mixed by gentle swirling, and equilibrated at room temperature for 1 h. MTX buffer concentrate was mixed with deionized water (1:15 *v*/v). Working solution of reagent A and B was prepared by diluting stock solutions 1:9 v/v with buffer solution.

Some calibrators (0, 0.20, 0.50, 1.00 μmol/L) were reconstituted to indicated concentration with 1.0 mL of deionized water according to the manufacturer’s instructions, other calibrators 1.50 and 2.00 μmol/L were reconstituted to 1.00 μmol/L with 1.50 and 2.00 mL deionized water respectively. Then the 0.20 μmol/L calibrator was diluted into 0.05 μmol/L, and the 1.00 μmol/L was diluted into 0.10 and 0.25 μmol/L. The new set of calibrators (0, 0.05, 0.10, 0.25, 0.50 and 1.00 μmol/L) meet the requirements of modified calibrator curve.

### Programming the Viva-E instrument

An open channel on the Viva-E was programmed to change volumes of reagents A & B from 180 μL to 110 μL (110 μL is the lower limit of Viva-E for MTX reagents). A six-point calibration curve was programmed with modified cubic spline regression. The six calibrators provided by the Kit were 0, 0.20, 0.50, 1.00, 1.50 and 2.00 μmol/L. When the volumes of reagents A and B were decreased, they were not sufficient to react with samples more than 1 μmol/L. As a result, the new calibrator set of modified EMIT assay was changed to 0, 0.05, 0.10, 0.25, 0.50, 1.00 μmol/L within a range of 0.05 to 1.00 μmol/L.

According to the instruction of the commercial kit, the samples of concentration more than 1.00 μmol/L should be diluted into the scope of calibration curve (0.05–1.00 μmol/L) using dilution factor of 10, 100 or 1000.

### Limit of blank, limit of detection, limit of quantitation

Limit of blank (LOB), limit of detection (LOD), limit of quantitation (LOQ) were determined according to CLSI EP17-A [4, 5]. LOB was assayed using six samples (S1–S6, 10 replicates over 10 days): S1 and S2 were zero calibrators from two different calibrator lots, S3 and S4 were diluent, S5 and S6 were blank plasma. LOD was assayed with 5 low-level sample pools ranging from the LOB to four times the LOB (12 replicates over 12 days). LOB and LOD were determined by two lots of reagents. According to the clinical requirements, the goal for total error was set to 20%. The test results of LOD study was used to estimate the bias and imprecision for each level of the analyte. Then these data were combined to estimate the total error at each level and determine the limit of quantitation (LOQ).

### Intra-day and inter-day imprecision

Intra-day (*n* = 10) imprecision of modified assay was evaluated by one level of control materials (0.05 μmol/L) and three levels of patient samples (0.12, 0.43, 0.82 μmol/L). At each level, 10 replicates were carried out. Inter-day (*n* = 25) imprecision was tested at these 4 levels in 5 consecutive days, and 5 replicates for each level. All the samples were obtained from different patients. The measured results were plotted against the expected values.

### Linearity

The linearity was verified by diluting the quality control of 2.00 μmol/L (hc) with buffer (c0) at the following ratios: 1hc + 1c0 (1.00 μmol/L), 2hc + 3c0 (0.80 μmol/L), 1hc + 2co (0.67 μmol/L), 1hc + 3co (0.50 μmol/L), 1hc + 5co (0.33 μmol/L), 1hc + 9co (0.20 μmol/L), 1hc + 19co (0.10 μmol/L), 1hc + 39co (0.05 μmol/L). Every dilution was analyzed three times, the linearity equation of modified assay was calculated based on the means of measured results and the expected values.

### The LC-MS/MS assay procedure

The LC-MS/MS assay performed in this study is as follows. Diphenhydramine was used as the internal standard. Acetonitrile was used as the serum precipitant to eliminate albumin. A Hypersil ODS-BP C18 (5 μm, 2.1 × 150 mm) column was used for LC separations. The mobile phase was 0.1% formic acid and acetonitrile. Gradient elution was performed at a flow rate of 0.5 mL/min at 30 °C. The multiple reaction monitoring (MRM) mode was used to detecte MTX and diphenhydramine in electrospray ionization (ESI) source and positive ion scan mode. MTX was detected at m/z 455.0 → 308.1, diphenhydramine was detected at m/z 256.0 → 167.0 [6, 7].

### Method comparison

Seventy-nine samples were collected from acute lymphoblastic leukemia patients in Hematological Ward of Dalian Children’s hospital. Every sample was tested using modified EMIT assay and LC-MS/MS assay respectively. The results were compared using simple linear regression analysis (MedCalc Statistical Software version 15.6.1). Another 45 samples were tested using modified EMIT assay and commercial EMIT assay for method comparison [8].

### Interference studies

Triglyceride interference was assessed by preparing samples with Intralipid (20% IV fat emulsion). The concentration of triglycerides in the samples was 1000 ng/dL. Then Paired-T test was performed.

Bilirubin interference was simulated using some MTX-free icteric plasma samples with bilirubin concentration no less than 30 mg/dL.

The interference from the structurally related metabolite 7-hydroxy-methotrexate was assessed by adding different levels of 7-hydroxy-methotrexate to the plasma samples, the concentration of MTX in those samples was 0.05 μmoL/L. And the test results of current samples were compared with the results of original samples [9, 10].

## Results

The LOB was found to be 0.0146 μmol/L and the LOD 0.0360 μmol/L. The data of LOD suggested CV% of pool measurements with concentration 0.0438 μmol/L was 9.82, and the estimation of total error (19.67) was less than the goal for total error. According to Total Therapy Study XVI for Newly Diagnosed Patients with Acute Lymphoblastic Leukemia (ALL), 0.05μmoL/L could meet the clinical requirements. Thus, LOQ was set to 0.05μmoL/L.

Intra-day imprecision (Table 1) was evaluated by within-run and inter-day imprecision (Table 2) between days. Inter-day imprecision data were collected by assaying the 4 levels (one control material, and three patient samples) 25 times on five consecutive days. Intra-day and inter-day imprecision showed good reproducibility at all levels (0.05, 0.12, 0.43, 0.82 μmol/L). It shows that the assay is reproducible at the low end.

**Table 1:** Intra-day Imprecision for Modified MTX Assay

Level	Target	Mean	SD	%CV	%Bias
1	0.05	0.050	0.004	9.43	< 0.01
2	0.12	0.121	0.010	8.22	0.83%
3	0.43	0.428	0.021	5.02	−0.47%
4	0.82	0.817	0.026	3.22	−0.37%

*N* = 10, Unit: μmol/L

**Table 2:** Inter-day Imprecision for Modified MTX Assay

Level	Target	Mean	SD	%CV	%Bias
1	0.05	0.049	0.006	11.62	−1.60%
2	0.12	0.113	0.011	9.78	−5.67%
3	0.43	0.418	0.019	4.55	−2.88%
4	0.82	0.799	0.032	4.11	−2.59%

*N* = 25, Unit: μmol/L

The analytical data for linearity was obtained from diluted quality control material with known concentration (1.00, 0.80, 0.67, 0.50, 0.33, 0.20, 0.10 and 0.05 μmol/L). The linearity equation of modified assay was y = 0.9913x + 0.0046, in which y was the mean of measured concentration and x was the target concentration (R^2^ = 0.9994).

LC-MS/MS assay also showed good linearity within the range of 0.002–2.20 μmol/L (1–1000 ng/mL). The linearity equation was Y = 0.0955X-0.026, R^2^ = 0.9971. The intra-day and inter-day RSD were both less than 9.0%. The average recovery of methotrexate was 95.86–112.31%.

**Fig. 1:**
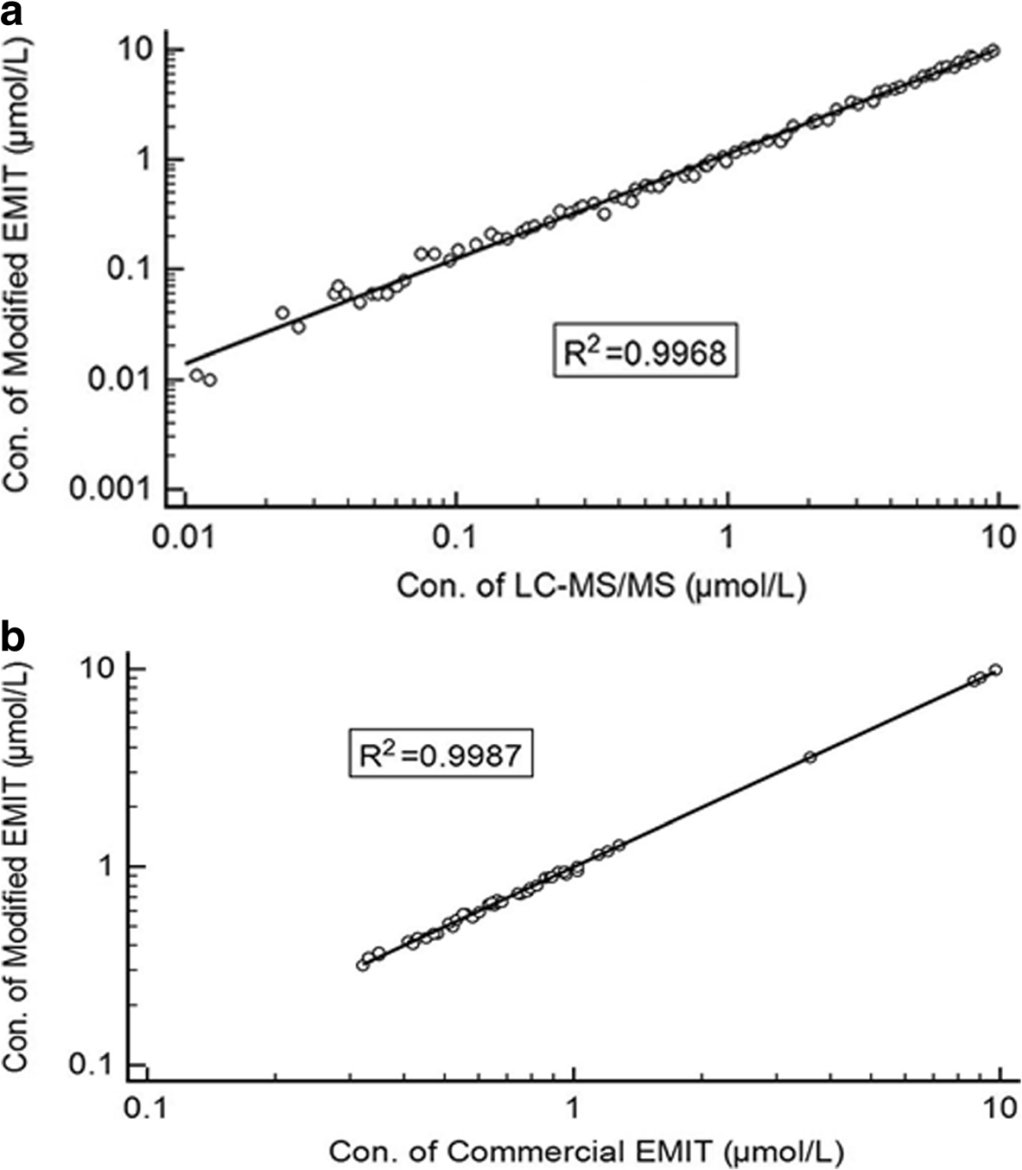
**a** Method comparison between modified EMIT assay and LC-MS/MS assay in the range of 0.05–10.00 μmol/L. (EMIT) = 1.0872(LC-MS/MS) + 0.0281, *N* = 79, R^2^ = 0.9968. **b**. Method comparison between Modified assay and Commercial assay in the range of 0.30–10.00 μmol/L. (Modified) = 1.0080(Commercial)-0.0064, *N* = 45, R^2^ = 0.9987

**Fig. 2:**
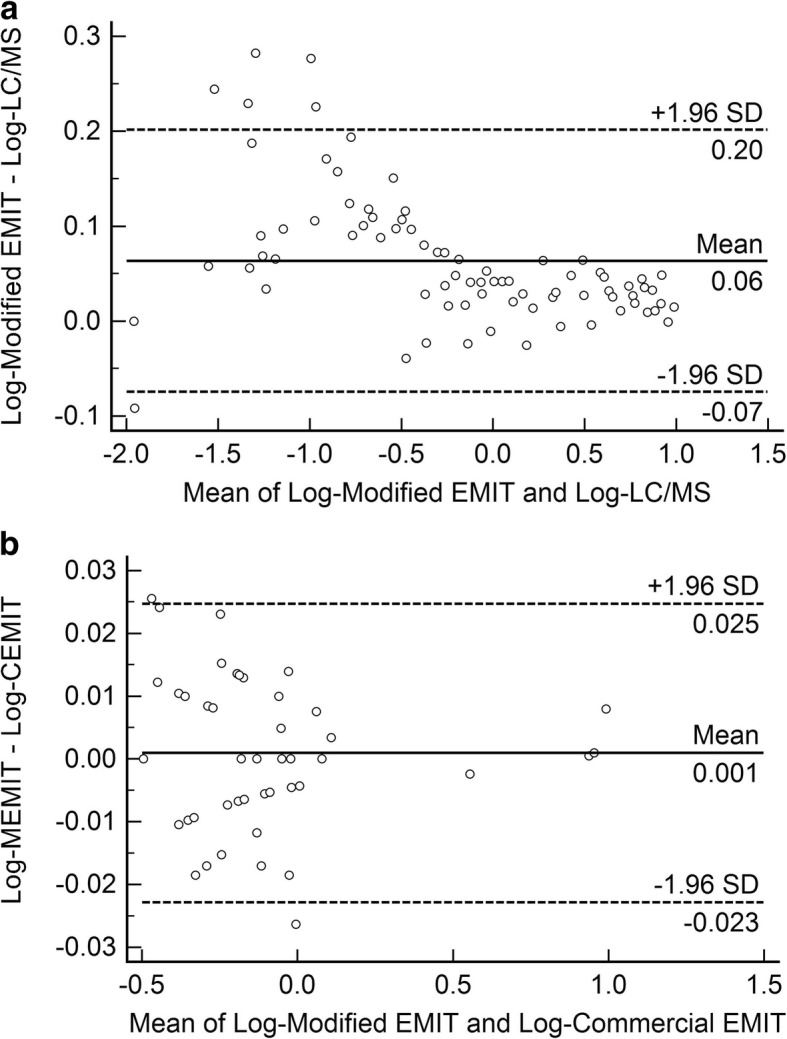
**a** Bland-Altman plot of the differences (Y-axis) and means (X-axis) of the logarithmic transformed values obtained from Modified EMIT and LC-MS/MS measurements (n = 79). **b**. Bland-Altman plot of the differences (Y-axis) and means (X-axis) of the logarithmic transformed values obtained from Modified EMIT and Commercial EMIT measurements (*n* = 45)

Method comparison was performed by comparing the results of modified EMIT assay with the results from LC-MS/MS assay and commercial EMIT assay respectively (Fig. 1a & 1b). For modified EMIT, the samples with concentration from 1.00 to 10.00 μmol/L should be diluted 10 times into the scope of calibration curve (0.05–1.00 μmol/L). In the range of 0.05–10.00 μmol/L, correlation between the Modified assay and LC-MS/MS was significant (*r* = 0.9968). In the range of 0.30–10.00 μmol/L, the correlation between modified and commercial assays was significant (*r* = 0.9987). As Fig. 2a and Fig. 2b shown, there was no significant difference between the results of LC-MS/MS assay and modified assay or between the results of modified and commercial assays.

Triglyceride and Bilirubin interference were simulated (Table 3), All Bias of three groups were less than 10%, which is an acceptable cutoff.

**Table 3:** Interference Studies

Sample	Mean	Bias	%Bias
Blank	0.56		
Triglyceride	0.61	0.05	8.93
Bilirubin	0.52	−0.04	7.14

*N* = 5, Unit: μmol/L

The interference of structurally related metabolite 7-hydroxy-methotrexate was also simulated (Table 4). The ratio of MTX to 7-hydroxy-methotrexate in the test samples ranged from 1:1 to 1:100. All Bias were less than 20%.

**Table 4:** Interference Studies of Structurally Related Compound

Estimated MTX (μmoL/L)	Estimated 7-hydroxy-methotrexate (μmoL/L)	Measured MTX (*n* = 5) (μmoL/L)	Bias	%Bias
0.05	0.00	0.049		
	0.05	0.506	0.0008	1.61
	0.50	0.522	0.0024	4.82
	1.00	0.543	0.0045	9.04
	2.50	0.566	0.0068	13.65
	5.00	0.597	0.0099	19.88

**Fig. 3:**
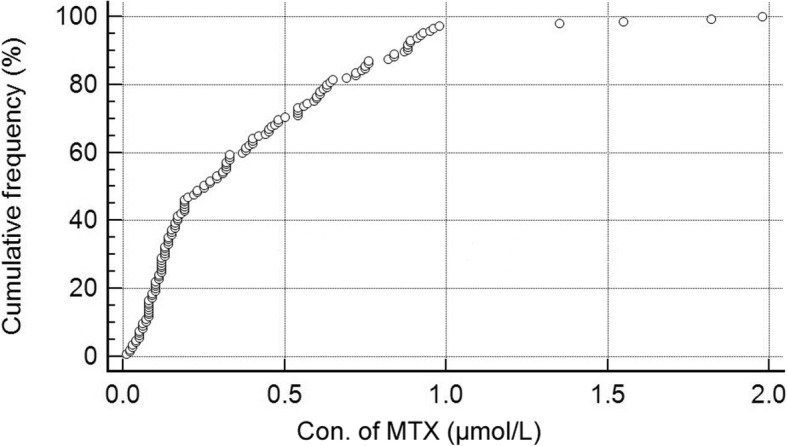
Distribution plot of MTX concentration in the range of 0–2.00 μmol/L, *N* = 145. X-axis represented the concentration of MTX, Y-axis represented the cumulative frequency (%) of every concentration levels. The cumulative frequency of concentration between 1.00–2.00 μmol/L was less than 3%

There was no obvious difference of dilution frequency between modified and commercial assays. The information of 202 samples were collected. Among them, 57 samples were collected at 23 h from the start of the infusion, they were more than 10.00 μmol/L and no need to be diluted in both two assays. The other 145 samples were collected more than 44 h, they were less than 2.00 μmol/L. Figure 3 shows a distribution plot of the 145 points, 141 points are under 1.00 μmol/L, 4 points are between 1.00–2.00 μmol/L. That means only 4 of the 202 samples need additional dilution to make their concentrations fall in the calibration range. Moreover, the test number of Reagent Kit increased from 140 to 210. As a result, the cost of detection was reduced about 30%.

## Discussion

There are two methods that can increase the sensitivity of EMIT MTX assay. The one is to increase the volume of samples. That has been reported. The other is to decrease the volume of reagents A and B. Both methods aim to increase the concentration of MTX in mixed solution for test. In this study, the volumes of reagents A & B were decreased from 180 μL to 110 μL, this procedure reduces the consumption of reagents and lowered the cost of the assay, so far it has not been issued by any thesis. Then the calibration set was modified by adding three low concentrations calibrators 0.05, 0.10, 0.25 μmol/L and removing 3 calibrators 0.20, 1.50, 2.00 μmol/L. The added calibrators were prepared by diluting manufacturer calibrators. As a result, an accurate assay with a measuring range of 0.05–1.00 μmol/L was established.

The comparison graph of modified EMIT and LC-MS/MS showed that the results of EMIT has a positive bias (Fig. 2), which is consistent with the published studies [11]. There were two possible influence factors, albumin-bound MTX and metabolite(s) of MTX found in plasma. However, the extraction efficiency of MTX in LC-MS/MS was 95.86%~ 112.31%, which means the acetonitrile precipitation is an effective approach to dissociate the bound drug from the protein. For this reason, bound MTX would not be the main factor that resulted in significant difference between the two methods. Maybe the metabolite of MTX caused the difference of the two methods.

In the following study, we aim to analyze the result of therapeutic MTX monitoring from childhood ALL patients in our hospital. Since the detection range of MTX extended from 0.3 μmol/L to 0.05 μmol/L, it enables us to establish a model for predicting MTX toxicity and delayed clearance, as well as to provide an accurate indicator for folinic acid rescue. This modified EMIT assay not just guarantees the rational and safety use of MTX, it also provides a more efficient way to conduct relative pharmaceutical or clinical studies.

## Conclusion

MTX has been used worldwide to treat childhood ALL in many protocols, it is necessary to detect the blood concentration of MTX at low level less than 0.3 μmol/L. This study modified the EMIT MTX assay on Siemens VIVA-E instrument to enhance the sensitivity from 0.3 μmol/L to 0.05 μmol/L. At the same time, the test number of a reagent Kit increased from 140 to 210, which means the cost of detection was reduced about 30%. The precision and correlation data indicate there is no difference between modified method and commercial method. The modified method could be used in routine clinical practice for monitoring MTX concentration at the level less than 0.3 μmol/L.

## Abbreviations

ALL: Acute Lymphoblastic Leukemia
CLSI: Clinical and Laboratory Standards Institute
EMIT: Enzyme Multiplied Immunoassay Technique
ESI: Electrospray Ionization
HDMTX: High Dose Methotrexate
LC-MS/MS: Liquid Chromatography-tandem Mass Spectrometry
LOB: Limit of Blank
LOD: Limit of Detection
LOQ: Limit of Quantitation
MRM: Multiple Reaction Monitoring
MTX: Methotrexate
QC: Quality Control
